# Supplementary figures and images for: Distribution patterns of Acidobacteriota in different fynbos soils
Source: PLoS One. 2021 Mar 22;16(3):e0248913. doi: 10.1371/journal.pone.0248913 (PMC7984625; doi:10.1371/journal.pone.0248913)

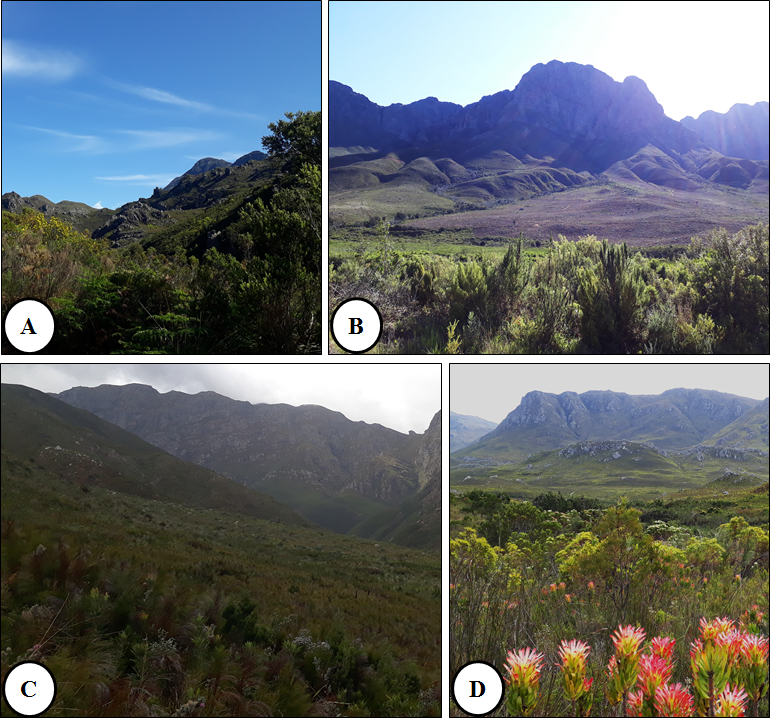

Supplement: S1 Fig — A. Hottentots Holland Nature Reserve, B-C. Jonkershoek Nature Reserve, and D. Kogelberg Nature Reserve. (TIF) [file pone.0248913.s001.tif]

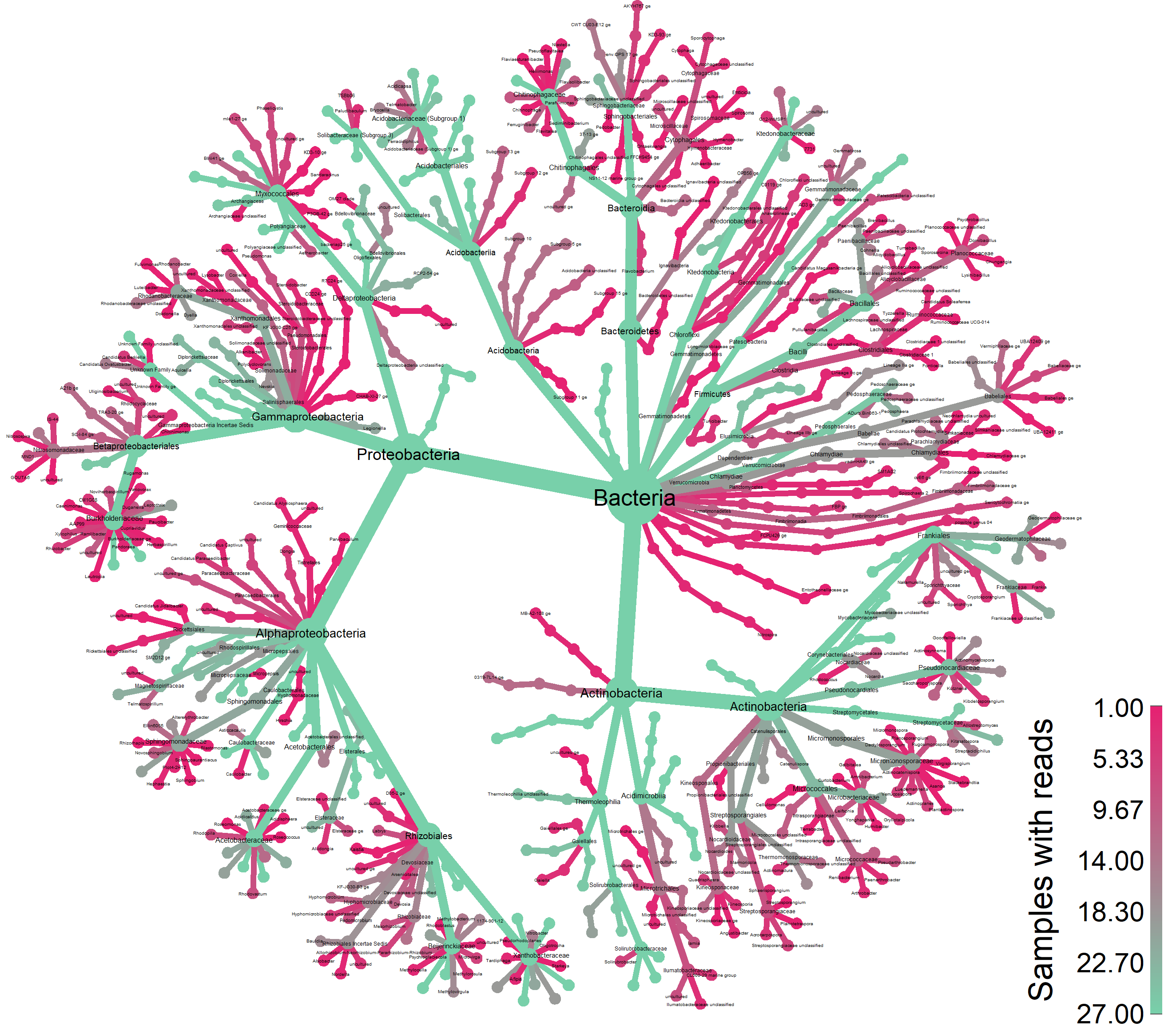

Supplement: S2 Fig — Node sizes indicate OTU count, with larger nodes representing greater OTU representation. Colours Green to Pink indicate the representation of each node in the different samples. Green–Detected in all samples. Pink–Detected in only one sample. (TIF) [file pone.0248913.s002.tif]
