# Supplementary material for: Distribution patterns of Acidobacteriota in different fynbos soils
Source: PLoS One. 2021 Mar 22;16(3):e0248913. doi: 10.1371/journal.pone.0248913 (PMC7984625; doi:10.1371/journal.pone.0248913)
Supplement: S1 Table — (PDF) [file pone.0248913.s003.pdf]

S1 Table. Sampling sites of three nature reserves and their fynbos and soil characteristics.

Site	Jonkershoek	Hottentots Holland	Kogelberg
**Latitude**	33° 59' 24.39" – 33° 59' 33.67" S	34° 2' 29.80" – 34° 3' 58.58" S	34° 17' 42.74" – 34° 19' 5.95" S
**Longitude**	18° 58' 44.36" – 18° 59' 44.53" E	19° 2' 7.70" – 19° 2' 44.15" E	18° 56' 20.59" – 18° 57' 48.74" E
**Fynbos types**	Kogelberg Sandstone<br>Cape Winelands Shale<br>Boland Granite	Kogelberg Sandstone<br>Coastal Shale<br>Boland Granite	Kogelberg Sandstone<br>Coastal Shale
**Soil type**	Clay-Loamy	Sand	Sand
