# Supplementary material for: Distribution patterns of Acidobacteriota in different fynbos soils
Source: PLoS One. 2021 Mar 22;16(3):e0248913. doi: 10.1371/journal.pone.0248913 (PMC7984625; doi:10.1371/journal.pone.0248913)
Supplement: S2 Table — (PDF) [file pone.0248913.s004.pdf]

S2 Table. Summary of the filtered and Acidobacteria-affiliated reads, together with the Acidobacteria relative abundance for each sample ID.

Sample Group	Sample ID	Filtered reads	Acidobacteria-affiliated reads	Acidobacteria relative abundance (%)
Jonkershoek	jaa	36,817	4,701	12.77
	jbb	38,390	1,315	3.43
	jd	25,685	1,308	5.09
	jdd	26,540	4,185	15.77
	je	34,291	3,289	9.59
	jf	28,619	2,683	9.37
	jg	43,914	4,284	9.76
	jh	28,093	2,374	8.45
	ji	33,427	2,271	6.79
	jj	33,357	3,050	9.14
Hottentots Holland	hb	32,548	5,730	17.60
	hc	31,819	2,927	9.20
	hd	31,056	5,585	17.98
	he	20,784	4,412	21.23
	hf	20,445	6,199	30.32
	hg	42,030	8,872	21.11
	hh	34,722	5,693	16.40
	hi	31,602	4,273	13.52
Kogelberg	ka	22,421	4,967	22.15
	kb	21,087	1,995	9.46
	kc	27,739	3,645	13.14
	ke	13,659	3,324	24.34
	kf	20,026	3,466	17.31
	kg	26,335	4,148	15.75
	kh	34,855	2,694	7.73
	ki	23,252	2,408	10.36
	kj	33,071	4,628	13.99
