# Supplementary material for: Distribution patterns of Acidobacteriota in different fynbos soils
Source: PLoS One. 2021 Mar 22;16(3):e0248913. doi: 10.1371/journal.pone.0248913 (PMC7984625; doi:10.1371/journal.pone.0248913)
Supplement: S3 Table — (PDF) [file pone.0248913.s005.pdf]

S3 Table. Analysis of Molecular Variance (AMOVA) statistical results of comparisons between nature reserves.

**Comparisons**	**F statistic**	***p*-Value**
Hottentots-Jonkershoek-Kogelberg	4.88	0.002*
Hottentots-Jonkershoek	5.85	0.021*
Hottentots-Kogelberg	2.23	0.131
Jonkershoek-Kogelberg	7.00	0.008*

A significant difference between community compositions is observed at $*p<0.05$.
