# Supplementary material for: Distribution patterns of Acidobacteriota in different fynbos soils
Source: PLoS One. 2021 Mar 22;16(3):e0248913. doi: 10.1371/journal.pone.0248913 (PMC7984625; doi:10.1371/journal.pone.0248913)
Supplement: S4 Table — (PDF) [file pone.0248913.s006.pdf]

S4 Table. Statistical evaluations of each soil abiotic variable and its contribution to the shift observed in the acidobacterial community using the corr.axes command in MOTHUR.

**Soil abiotic variable**	***p*-Value**	**Significance**
pH	< 0.001	***
H	0.91	-
P	0.002	**
K	0.02	*
Na.(EC)	0.24	-
K.(EC)	0.012	*
Ca.(EC)	0.88	-
Mg.(EC)	0.60	-
C	0.45	-
Na.bs.	0.16	-
K.bs.	0.005	**
Ca.bs.	0.82	-
Mg.bs.	0.14	-

A significant difference is observed at * $p<0.05$; ** $p<0.01$; *** $p<0.001$.

EC–exchangeable cations

bs–base saturation
