# Supplementary material for: Distribution patterns of Acidobacteriota in different fynbos soils
Source: PLoS One. 2021 Mar 22;16(3):e0248913. doi: 10.1371/journal.pone.0248913 (PMC7984625; doi:10.1371/journal.pone.0248913)
Supplement: S5 Table — Spearman’s rho (ρ) correlation coefficient is given. (PDF) [file pone.0248913.s007.pdf]

S5 Table. Spearman correlation test results for correlations between acidobacterial SDs to soil abiotic properties. Spearman's *rho* (ρ) correlation coefficient is given.

Soil abiotic property	SD1	SD1 Unclassified	SD2	SD3	SD3 Unclassified	Other[1]
**pH**	**-0,404**	0,213	**0,505**	-0,161	0,143	**0,438**
**H**	**-0,424**	0,102	0,010	0,134	**0,430**	-0,066
**P**	-0,342	0,257	**0,485**	-0,211	0,127	0,326
**K**	**-0,475**	0,310	**0,435**	-0,207	**0,398**	**0,432**
**C**	**-0,432**	0,152	0,134	0,015	**0,408**	0,082
**K.(EC)**	**-0,488**	0,323	**0,463**	-0,217	0,378	**0,429**
**K.bs.**	-0,344	0,322	**0,523**	-0,342	0,184	**0,433**
**Na.(EC)**	-0,141	0,205	0,267	-0,268	0,076	0,025
**Na.bs.**	-0,113	0,102	0,328	-0,126	-0,122	0,188
**Ca.(EC)**	0,039	0,209	0,147	-0,354	0,037	-0,107
**Ca.bs.**	0,189	0,139	0,063	-0,328	-0,056	-0,164
**Mg.(EC)**	-0,014	0,185	0,015	-0,220	0,272	-0,081
**Mg.bs.**	0,337	0,005	-0,171	-0,191	0,015	-0,067

[1]Other include all subdivisions with relative abundances < 1%.

A significant correlation is observed at $p<0.05$ and is indicated in bold red. SD1 Unclassified and SD3 Unclassified contain Acidobacteria OTUs with no taxonomic classification at genus level.

EC–exchangeable cations

bs–base saturation
