# Supplementary material for: Distribution patterns of Acidobacteriota in different fynbos soils
Source: PLoS One. 2021 Mar 22;16(3):e0248913. doi: 10.1371/journal.pone.0248913 (PMC7984625; doi:10.1371/journal.pone.0248913)
Supplement: Attachment — Submitted filename: Response to reviewers.docx [file pone.0248913.s008.docx]

First, we would like to thank the reviewers for their time and appreciate their input. Below is our response (in red) to the comments made by the reviewers.

**Reviewer #1:**

Minor issues:

Abstract and elsewhere – Acidobacteriota is the current accepted nomenclature - Whitman WB, Oren A, Chuvochina M, da Costa MS, Garrity GM, Rainey FA, Rossello-Mora R, Schink B, Sutcliffe I, Trujillo ME, et al. Proposal of the suffix -ota to denote phyla. Addendum to 'Proposal to include the rank of phylum in the International Code of Nomenclature of Prokaryotes'. Int J Syst Evol Microbiol 2018; 68:967-969

The nomenclature has been corrected to Acidobacteriota in the abstract and the rest of the manuscript.

Line 40 – You do not report new strategies for isolation of new microbes, this sentence is misleading.

This sentence has been removed from the abstract.

Line 58- includes

Corrected in text.

Line 103 – Were the samples of each type of soil mixed together or each was treated as a replicate? It would be good to have this described.

A sentence has been added to describe how we treated each sample:

L106-107 - ‘Each sample collected was treated as a replicate for the different nature reserves.’

Line 125 – missing the references of both primers

Reference has been added. The same primers were used as indicated by Postma et al. 2016.

Line 169-174 – There is no need to repeat the numbers that are in the table.

These numbers have been removed.

Line 333 – where, instead of were

Corrected in text.

**Reviewer #2**

Line 54. Could the authors find another word for "proliferation"? In microbiology this word implies growth.

Proliferation has been changed to ‘prevalence’.

Line 49. (…) 16S rRNA gene (as properly written in line 186).

This has been corrected in text.

Line 77. It would be nice to state the relative abundance of Acidobacteria previously found in Fynbos habitats (there are many studies cited here, and this information can only be found in the discussion section).

This information has been added to the introduction. See lines 78-80.

Line 208. 55% of the community or of the Acidobacteria community?

Clarified to ‘55% of the acidobacterial community’.

The Discussion needs some rewriting, it is mostly repletion of the results text.

The discussion has been changed to the best of our understanding to avoid repletion of the results.

In the discussion section of SD2 (lines 294- 320), the authors may consider add the information in reference: Catão, Elisa CP, et al. " International journal of microbiology 2014 (2014). They did a similar discussion on subgroup 2

We appreciate suggesting this article. The discussion has been revised accordingly and the reference added. See lines 303-305 and 317-320.
